# Vietnam’s Horse Sector: A Comprehensive Review of History, Production Systems, Health Challenges, and Research Priorities

**DOI:** 10.3390/ani16132015

**Published:** 2026-07-01

**Authors:** Van Thanh Nguyen, Nguyen Van Ba, Nguyen Van Dai, Lan Doan Pham, Duy Ngoc Do

**Affiliations:** 1 Faculty of Veterinary Medicine, Viet Nam National University of Agriculture, Hanoi 100000, Vietnam; thanhnv1102@gmail.com; 2 Key Laboratory of Animal Cell Technology, Vietnam Institute of Animal and Veterinary Sciences, Thuongcat, Hanoi 100000, Vietnam; nguyenba81@yahoo.com (N.V.B.); pdlanvn@yahoo.com (L.D.P.); 3 Mountainous Animal Husbandry Research and Development Center, Thai Nguyen 256460, Vietnam; daibavan60@gmail.com; 4 Institute of Research and Development, Duy Tan University, Da Nang 550000, Vietnam; 5 School of Medicine and Pharmacy, Duy Tan University, Da Nang 550000, Vietnam; 6 Department of Animal Science and Aquaculture, Dalhousie University, Truro, NS B2N 5E3, Canada

**Keywords:** horse, Vietnam, native breeds, equine health, working equids, socio-cultural roles

## Abstract

Horses in Vietnam have a long historical presence and continue to hold economic, socio-cultural, and medicinal importance across diverse regions. Despite these roles, comprehensive scientific information on equine development, including health and disease patterns, reproductive performance, genetic resources, and biomedical applications, remains limited and fragmented. In this review, we summarize existing knowledge on equine populations, production systems, and their development in Vietnam, as well as their roles in cultural practices, livelihoods, and traditional medicine. We then identify major gaps in surveillance, genetic characterization, and coordinated research capacity by synthesizing evidence from breeding, health, reproduction, and emerging research areas. Finally, we highlight where current efforts fall short and outline key priorities for future work to strengthen equine science, support culturally relevant practices, and guide the sustainable development of Vietnam’s horse sector. Overall, the synthesis information shows (i) native Vietnamese horses belong to the Southeast Asian pony complex and include at least four recognized populations; (ii) the northern mountain provinces account for the substantial majority of the national herd; (iii) strongyle infections, Trypanosomiasis, haemorrhagic septicaemia and the recently confirmed equine piroplasmosis are the principal health challenges; and (iv) genetic characterization, reproductive management, welfare assessment, and capacity building are the four most critical research priorities identified. This information is important for prioritizing future research and conservation activities and enhancing the development of Vietnam’s horse population.

## Simple Summary

Horses are a part of daily life in many provinces in Vietnam. They are used for transport, farming, cultural traditions, and medical purposes. However, fragmented information on the breeds, production, health, breeding, and diseases in the country hinders the development and use of the species. In this review, we summarize current knowledge of horses in Vietnam, including their history, social and economic importance, and the health challenges they face. We also discuss how horses are bred and what research has already been done on reproduction, genetics, and biomedical uses. By identifying gaps in knowledge, we highlight several areas where future research is especially needed, such as better disease monitoring, improved breeding methods, and implementation of government support and policy. Our goal is to provide a clear overview that can help guide future studies and support the sustainable development of Vietnam’s horse population.

## 1. Introduction

Horses have played a modest but persistent role in Vietnam’s ecological, cultural, and socio-economic landscape [1]. Although the country is not traditionally recognized as a major equine center compared to Mongolia or China, historical and ethnographic evidence shows that horses have been present across multiple regions of Vietnam for centuries. Their origins largely trace to Mongolian-type horses, which were introduced through long-standing interactions between northern Vietnamese ethnic groups and populations across the Sino-Tibetan frontier. These horses are often referred to as Hmong or Vietnamese native horses. These horses belong to the broader Southeast Asian pony complex, a genetically and ecologically coherent group of small, hardy mountain ponies distributed across southern China, Laos, Myanmar, and Vietnam. They are characterized by small stature, high endurance, and the ability to survive on low-quality forage.

Horses serve both practical and symbolic functions in Vietnam. They are used for transport, pack work, and mobility in upland regions, where rugged landscapes limit the use of mechanized vehicles. These upland regions form part of the Northern Mountainous Zone, an area spanning roughly one-third of Vietnam’s total landmass and home to over 30 officially recognized ethnic minority groups, many of whom maintain horses as a central element of their material culture, transport economy, and seasonal livelihoods. Horses also played a role in military logistics during various Vietnamese dynasties. The cultural significance of horses is evident in Vietnamese folklore, religious iconography, and traditional art [2]. For example, horses appear in Dong Ho folk paintings, where they symbolize loyalty, nobility, and scholarly achievement, reflecting a deep cultural association [3].

At present, Vietnam’s horse population remains relatively small compared to cattle, buffalo, or pigs, but horses continue to hold economic and cultural relevance in specific regions. Indigenous horses are valued for their hardiness and low maintenance requirements, making them suitable for smallholder systems in mountainous provinces. Their role in tourism, such as in Bac Ha’s ethnic markets, has also grown [4]. However, systematic scientific research on Vietnamese equine genetics, population structure, and conservation status remains limited. As Vietnam undergoes rapid rural transformation by shifting in labour practices, mechanization, and tourism, the role of horses is changing [5]. The limited research base reflects broader challenges in veterinary research capacity and institutional support for equine studies in Vietnam. Nevertheless, recent investigations have begun to document important parasitic diseases and cultural dimensions of horse-keeping in Vietnam, as discussed in Section 4.2 and Section 5.3 of this review. Understanding the current state of knowledge, identifying critical gaps, and establishing research priorities are essential steps toward supporting sustainable equine health and industry development in Vietnam.

This review therefore aimed to (i) synthesize available evidence on the historical development, population structure, and production systems of horses in Vietnam; (ii) characterize their economic, socio-cultural, and biomedical roles; (iii) assess the current state of equine research in the country across breeding, reproduction, and health; and (iv) identify priority areas and a strategic agenda for future research and development. To our knowledge, this represents the first English-language synthesis of Vietnamese equine science, bringing together locally embedded, non-indexed, and official and peer-reviewed sources into a coherent international framework.

## 2. Methods

Due to a lack of references or studies related to Vietnamese horses from major databases, this review synthesizes evidence through expert-guided, broad, and iterative searching across multiple source types. The literature was identified through searches of the following databases and repositories: PubMed/MEDLINE, Web of Science (Core Collection), Scopus, Google Scholar, CAB Abstracts, and FAO AGRIS. Searches were conducted between September 2025 and December 2025. English-language search terms used across databases included combinations of the following: ‘horse’, ‘equine’, ‘equid’, ‘Vietnam’, ‘native breed’, ‘genetic resources’, ‘crossbreeding’, ‘Kabardin’, ‘White Horse’, ‘working equid’, ‘welfare’, ‘strongyle’, ‘Trypanosoma evansi’, ‘piroplasmosis’, ‘serum production’, ‘antivenom’, and ‘Southeast Asia’. Because Vietnamese equine science is sparsely represented in international databases, returning only a small number of records across all English-language databases combined, we also performed additional searching through Vietnamese-language sources, including the portal of the Vietnamese Ministry of Agriculture and Rural Development, the institutional repositories of the National Institute of Animal Sciences (NIAS) and Thai Nguyen University of Agriculture and Forestry, and Vietnamese academic journals accessible through the Vietnam National Library digital portal. Vietnamese-language search strings included: “ngựa Việt Nam” (horse + Vietnam), “ngựa nội” (Vietnamese local horse breeds”), “nghiên cứu ngựa” (horse research), ngựa bạch (White Horse), “giống ngựa bản địa” (native horse breed), “bảo tồn nguồn gen ngựa” (horse genetic resource conservation), “chăn nuôi ngựa miền núi” (horse husbandry in mountainous areas), and “ngựa H’Mông” (H’Mong horse). These terms were entered individually and in combination; no Boolean operator restrictions or date filters were applied.

The yield of peer-reviewed, English-language results specifically addressing Vietnamese horses was very small across all major databases, consistent with the fundamental premise of this review that equine science in Vietnam is understudied and fragmented. The majority of sources incorporated in this review were identified through: (i) database searching as described above; (ii) manual reference tracking from retrieved articles; (iii) direct consultation of Vietnamese institutional reports and grey literature held by co-authors with professional affiliations to Vietnamese equine research institutions; and (iv) targeted searches for named authors and research projects known to the author team through professional networks.

Sources were included if they contained primary data, substantive descriptive information, or policy and technical guidance relevant to one or more of the following topics in the Vietnamese context: horse population structure and breeds; historical roles and socio-cultural significance; production systems and livelihoods; breeding, crossbreeding, and genetic improvement; reproductive physiology and management; health, parasitology, and disease; animal welfare; biomedical applications; and priorities for future research and development. Sources were excluded if they were entirely unrelated to the Vietnamese equine context or duplicated information already captured from a higher-quality primary source. Notably, grey literature, including institutional research reports, provincial veterinary records, Vietnamese-language journal articles, government livestock development plans, and news reports from credible Vietnamese media, was included where it represented the only available source for a specific claim. This decision reflects the reality that a substantial proportion of Vietnamese equine research has not been published in international peer-reviewed journals; excluding grey literature would have produced a severely incomplete and distorted picture of the available evidence. Finally, evidence was synthesized narratively and organized thematically in accordance with the review objectives stated. Quantitative data from multiple sources were reported as ranges or central estimates with explicit source attribution. No formal risk-of-bias assessment was applied, as the majority of included sources are descriptive or observational in nature and do not present comparative experimental data amenable to standardized quality appraisal.

## 3. Historical Development and Current Structure of Horse Populations in Vietnam

### 3.1. Historical Roles of Horses in Vietnam

**Figure 1:**
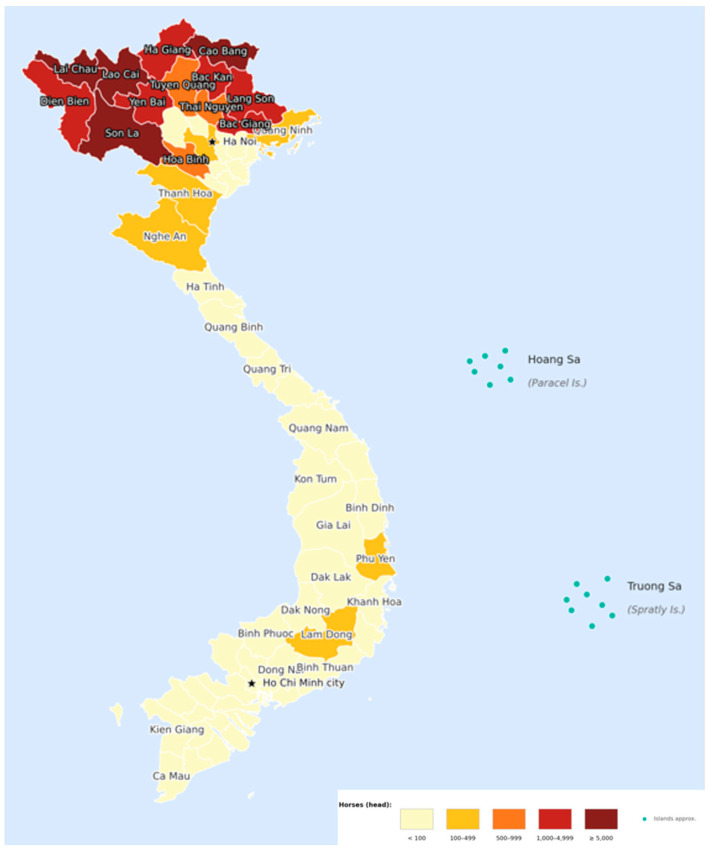
Distribution of horses in different provinces in Vietnam.

Horses have a long history in Vietnam, but they are not a major livestock species in the country. Yet, they remain particularly important in upland regions, where they played an essential role in daily life. Before the expansion of paved roads and mechanization in the 1990s and 2000s, horses were one of the most reliable means of transport in the northern mountainous zone, which is an area spanning roughly one-third of Vietnam’s total landmass, especially in provinces such as Lao Cai, Ha Giang, Cao Bang, and Bac Kan, as well as in other rocky regions in the Central and Highland regions. Currently, they are very useful in some regions where the terrain is steep, fragmented, and often isolated, and where road access remains limited. Their distribution by province is shown in Figure 1.

For local ethnic communities, especially the H’Mong, Tay, and Nung, horses are used to carry maize, rice, salt, firewood, medicinal plants, and household goods along narrow mountain paths [6]. They also transported people between villages and markets, supporting small-scale trade networks that connected remote communes to district centers.

Horses also contributed to military logistics in earlier periods. Historical documents from the French colonial era and earlier dynasties show that horses were used to move supplies, messages, and equipment in mountainous campaigns. Although Vietnam did not develop a strong cavalry tradition like Mongolia or northern China, horses still supported transport and communication in difficult terrain [7]. Their endurance and ability to survive on low-quality forage made them suitable for long journeys across rugged landscapes.

Culturally, horses appear in several traditional activities [6]. For example, the annual horse racing festival is a well-known cultural event where local riders compete on native horses without saddles in Lao Cai province [4]. The festival symbolizes strength, pride, and community identity, and it attracts both domestic and international visitors. Horses also appear in Vietnamese folk art, where they represent loyalty, status, and scholarly success [8]. These cultural expressions show that horses are not only working animals but also symbols of social value and local heritage.

### 3.2. Population Dynamics and Regional Distribution

**Figure 2:**
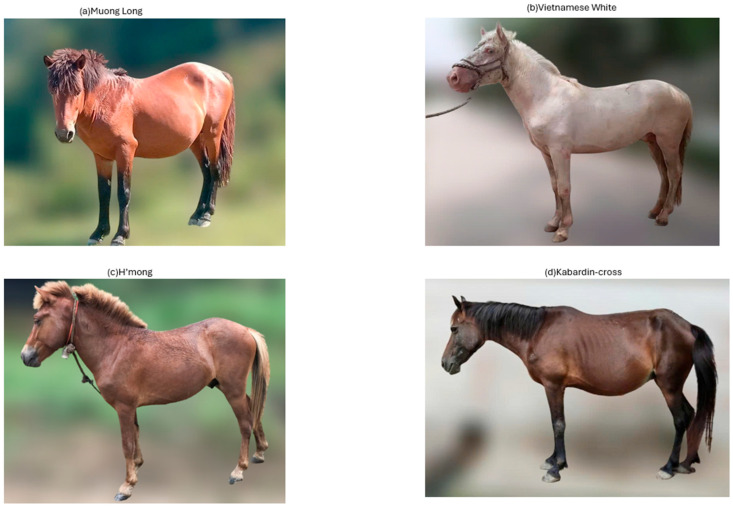
Phenotypic variation among local and crossbred horse populations in Vietnam. Panels illustrate standard conformation for (**a**) Muong Long, (**b**) Vietnamese White, (**c**) H’mong, and (**d**) Kabardin-cross.

Vietnam’s horse population has changed significantly over the last three decades. According to the General Statistics Office (GSO), horse numbers were relatively high in the early 1990s (approximately 130,000–140,000 animals nationally, GSO data), when many upland households still depended on horses for transport. Vietnam’s native horse populations are primarily small, hardy mountain ponies belonging to the broader Southeast Asian pony complex, specifically adapted to rugged terrain and humid climates. There are several horse breeds or populations raised in Vietnam, including Hmong, Muong Luong, Vietnamese White horse and Improved Kabardin-cross. The Hmong (or Mong) horse is the most widely distributed, standing roughly 1.1–1.3 m and weighing approximately 200 kg. These animals are characterized by their sturdy conformation, hard hooves, and “low-input” resilience, allowing them to thrive on natural grazing and crop residues while carrying heavy loads on steep mountain trails [9]. A specialized subset is the Muong Long horse of the Nghe An highlands; while physically similar to other native types, it is distinct for its adaptation to extreme altitudes (above 1300 m) and its deep cultural ties to the Hmong “heaven gate” communities [10]. Beyond these standard types, phenotypic and genetic variations offer specialized utility. The Vietnamese White horse maintains the general conformation of native ponies but is prized for its consistent white coat [1,11]. To address the limitations of the native ponies’ small stature, the Improved Kabardin-cross has been introduced. The Kabardin is a hardy mountain breed originating from the Caucasus region of Russia, historically valued for its endurance, sure-footedness at altitude, and resistance to harsh climatic conditions. These characteristics have been developed through centuries of use as a pack and military transport horse across steep terrain, making it a well-suited outcross for improving the load capacity of Vietnamese native ponies while retaining their mountain adaptability. By crossing local mares with Kabardin blood (typically 25%), breeders have produced hybrids with 30–50% greater carrying capacity than pure indigenous types. Despite these introductions, the native breeds remain essential symbols of local identity and are prized for their superior resistance to tropical diseases and their ability to navigate paths too narrow or steep for larger, imported breeds (Table 1). Representative photographs of each breed type are shown in Figure 2.

**Figure 3:**
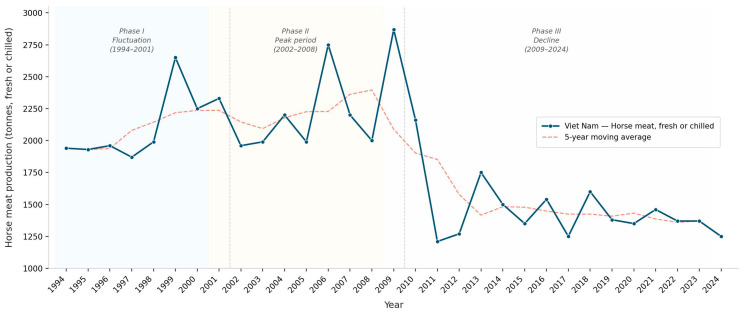
Horse meat production (tonnes, fresh or chilled) in Vietnam over the years (Source: FAO, 2026 data, FAOSTAT [12] (accessed March 2026)).

Based on FAO data [12], Vietnam’s horse population expanded steadily from the early 1960s to the late 1970s, rising from about 49,000 to more than 140,000 animals, a pattern mirrored by increasing slaughter numbers and meat production during the same period, as horses remained important for transport, agriculture, and local food supply. In Vietnam, horses are slaughtered as a source of food in some upland communities, where horse meat is a culturally valued product sold at ethnic markets. Slaughter numbers, therefore, broadly co-vary with population size and working horse utilization, making them a useful, even if imperfect, proxy for trends in the working horse sector. After 1979, population size and slaughter volumes began to fluctuate, with several rebounds but no return to earlier peaks, although 1999 marked a brief surge in both horse numbers and meat output. Through the 1980s and 1990s, slaughter ranged between roughly 9000 and 18,000 animals per year, while the national herd stabilized at intermediate levels, suggesting a balance between continued rural use and gradual mechanization. From the early 2000s onward, the national horse population declined consistently, falling to around 46,000 by 2024, and slaughter numbers followed a similar downward trajectory. After 2010, total meat production declined gradually from approximately 1500 t to roughly 1250 t by 2024, a 17% reduction over 14 years, driven primarily by decreasing slaughter numbers rather than changes in individual carcass weight, and reflecting the broader structural contraction of the working horse sector as mechanization expanded (Figure 3).

Although the equine sector in Vietnam is small, it includes three distinct production systems. These systems differ in herd size, management practices, feeding strategies, labour organization, and market orientation, including (1) smallholder mountain systems, in which ethnic minority households maintain horses primarily for pack transport and household capital; (2) state research and breeding centres, which focus on conservation, genetic improvement, and agricultural extension services; and (3) private and commercial enterprises, which have emerged in response to growing demand for sport and leisure and tourism activities horses.

#### 3.2.1. Smallholder Mountain Systems

In Vietnam’s upland regions, especially in the northern mountains, horses remain closely linked to the livelihoods of ethnic minority households. These smallholder systems are typically found among H’Mong, Tay, Nung, Dao and other communities living in steep, fragmented landscapes where mechanized transport is limited [13,14]. In these areas, horses continue to provide essential transport services. They carry maize, cassava, fuelwood, bamboo, medicinal plants, and household goods over long distances, often along narrow mountain paths that connect remote villages to commune centres and weekly markets. This role is especially important during harvest seasons, when households must move large volumes of maize or cassava from upland fields to storage or sale points. In many villages, a single horse may travel over distances of 5–20 km per day on typical market routes, and occasionally up to 30–40 km for seasonal trading journeys between remote communes and district centres, often along mountain paths that are inaccessible to motorized vehicles to support both household subsistence and small-scale trade.

Grazing practices in these systems are simple and rely heavily on natural resources. Horses graze on natural pastures, forest margins, fallow fields, and roadside vegetation, depending on the season [15]. During the rainy season, forage is abundant, and horses can maintain body condition with minimal supplementation. However, in the dry and cold months, especially from December to February, feed shortages are common. Households then rely on crop residues such as maize stover, rice straw, and cassava leaves. These feeds are low in protein and energy, so horses often lose weight during winter. Supplementation with bran or concentrate is rare due to cost constraints. Water access also varies seasonally, with some households leading horses to streams or communal water points once or twice per day.

Horses are integrated into broader mixed farming systems that include buffalo, cattle, pigs, chickens, and goats [16]. Buffalo and cattle provide traction and manure, while pigs and poultry contribute cash income [17]. Horses complement these species by providing mobility and transport, allowing households to move inputs and outputs across difficult terrain. In many upland communities, a horse is considered a key household capital asset, similar to a buffalo [14]. It represents stored wealth, insurance against shocks, and a means to access markets. When households face urgent expenses, such as medical costs, school fees, or crop failure, they may sell a horse to generate cash. This economic function explains why some families maintain horses even when mechanized transport becomes available.

#### 3.2.2. Research and Breeding Centres

Vietnam maintains several state-run equine research and breeding centres, the most prominent being the Mountain Livestock Research and Development Centre [18]. Established during the mid-20th century, this centre was originally mandated to conserve local horse genetic resources, improve performance traits, and test new genotypes suitable for mountainous regions. Over time, its mandate expanded to include breeding, conservation, performance testing, pasture management research, and the production of hyperimmune plasma and serum-based biological therapeutics, including antitoxins and antivenoms. These functions are documented in national livestock development plans and research reports from the Vietnam Academy of Agricultural Sciences (VAAS). The centre maintains structured herds consisting of local Vietnamese horses, crossbred lines, and occasionally specialized imported breeds used for testing. Herd sizes vary by year; historical reports from Ba Van indicate a maintained population of approximately 200–400 horses during active breeding periods, with the total declining in recent decades as funding constraints reduced the scale of operations [18]. Pasture management is more organized than in smallholder systems, with rotational grazing, forage plots, and supplementary feeding during dry seasons. Horses are selected based on body size, conformation, docility, reproductive performance, and work capacity, reflecting both traditional and modern breeding objectives. One of the centre’s important roles is extension and genetic improvement. For decades, it has supplied stallions and crossbred foals to cooperatives and households in surrounding provinces such as Son La, Hoa Binh, Lao Cai, and Ha Giang [18]. Historical reports indicate that thousands of crossbred foals have been distributed through these programs, contributing to the improvement of local horse populations. The centre also provides technical services, including training on feeding, disease prevention, and reproductive management. These activities help maintain the genetic base of Vietnamese horses while supporting rural development goals. Other state units, including provincial livestock research stations, also maintain smaller horse herds for conservation and breeding. Although their scale is more limited, they contribute to maintaining genetic diversity and supporting local production systems.

#### 3.2.3. Private and Commercial Enterprises

In recent years, Vietnam has seen the emergence of private and commercial equine enterprises, reflecting changing consumer demand and the growth of tourism and sports activities. These farms often invest in imported sport horses, including European breeds used for racing, show jumping, dressage, and high-end tourism services. Such enterprises are found in provinces like Lao Cai, where tourism is expanding, as well as in peri-urban areas around Ha Noi and in Da Lat, Lam Dong province, where scenic landscapes attract visitors. Investment levels in these systems are high. Farmers may spend 100–200 million Vietnamese Dong (or more) per breeding animal, and additional capital is required to build stables, training tracks, riding arenas, and tourism facilities [19,20]. Feeding practices rely on purchased concentrate, high-quality forage, and veterinary care tailored to sport horses. Labour is specialized, with trained handlers and riding instructors. These systems are capital-intensive and exposed to market risks, including fluctuations in tourism demand, disease outbreaks, and limited technical support for managing sport horses in tropical environments. Despite these challenges, the sector continues to grow, driven by rising interest in equestrian sports and experiential tourism.

## 4. Economic, Socio-Cultural and Medical Roles of Horses in Vietnam

Across Vietnam, horses serve multiple functions that reflect both traditional practices and modern economic opportunities (Table 2). In many upland communities, horses remain essential for work and transport, especially in areas where roads are limited. They carry agricultural products, household goods, and sometimes people across long distances. In peri-urban areas, small numbers of horses are used for carriage services, transporting goods or tourists along short routes.

### 4.1. Livelihood and Economic Functions

Horses continue to serve practical functions in Vietnamese rural livelihoods, particularly in mountainous and remote areas where mechanization may be limited. Evidence from livestock development programs indicates that horse production is included in farmer group interventions aimed at enhancing smallholder production and market access in districts such as Mai Son in Son La Province. This inclusion suggests that horses retain economic value for working functions, potentially including agricultural labour, transportation, and income generation [21]. The integration of horses into livestock development initiatives reflects recognition by development practitioners of their continued relevance to rural household economies. However, the specific economic contributions of horses, including their roles in agricultural production, transportation, breeding, or sale, remain poorly documented in the scientific literature. The scale of the equine population, market values, income contributions, and trends in horse ownership are not quantified in available studies.

### 4.2. Cultural Symbolism and Linguistic Representation

Horses occupy a place in Vietnamese cultural expression, as evidenced by their presence in idiomatic language. Within Vietnamese culture, horses are most prominently associated with strength, perseverance, and scholarly achievement, symbolism visible in Dong Ho folk paintings, zodiac traditions, and the prestige surrounding native horse racing festivals such as the Bac Ha event in Lao Cai, while simultaneously serving as markers of ethnic minority identity and material wealth in upland communities. Comparative linguistic analyses of animal-related idioms reveal that while Western cultures prominently valorize horses as symbols of speed and strength, Vietnamese idiomatic usage reflects different patterns of animal salience. This suggests that horses, while present in Vietnamese cultural language, may not hold identical symbolic weight as in Western or Central Asian cultures, where horses have been central to nomadic traditions and military power [22]. The cultural symbolism of horses in Vietnam appears to be shaped by the country’s agricultural rather than pastoral heritage, with water buffalo and other livestock potentially holding greater cultural prominence. Nevertheless, the presence of horse-related idioms indicates historical and ongoing cultural awareness of horses and their characteristics. The specific meanings, contexts, and regional variations in horse symbolism within Vietnamese culture warrant further ethnographic investigation [23].

Historical records also document colonial and post-colonial efforts at horse breeding in Vietnam, suggesting that horses have been subjects of state attention and institutional development efforts, albeit with limited success [5]. These historical breeding programs imply that horses were valued for military, transportation, or agricultural purposes during different periods of Vietnamese history. Beyond their physical labour contributions, horses hold deep cultural meaning, such as in festivals and rituals [4,24], symbolic value, social status, and tourism. Understanding these cultural dimensions is essential for designing conservation strategies that respect community traditions.

### 4.3. Equine-Derived Sera in Vietnam’s Health System

Horses play a strategic biomedical role in Vietnam as living bioreactors for hyperimmune plasma used in antitoxins and antivenoms. The rationale for using horses in serum production is grounded in immunobiology: their large body size and blood volume, capacity to generate high-titer antigen-specific antibodies after controlled hyperimmunization, and tolerance of repeated plasma collection make them the preferred species for large-scale antiserum manufacture [25]. The Suoi Dau horse breeding farm in Khanh Hoa, established in the late nineteenth century by Alexandre Yersin as part of his serum-production work and now managed by the Institute of Vaccines and Medical Biologicals (IVAC) [20], maintains approximately 300–400 purpose-raised horses under controlled husbandry for this purpose. Each year, the farm supplies on the order of 10,000–12,000 litres of crude plasma that are processed into anti-tetanus, anti-diphtheria, anti-rabies, and snake-antivenom products, including Vietnam’s first domestically produced cobra and pit-viper antivenoms, which have expanded access to life-saving treatment in rural areas where venomous snakes are common and imported antivenoms were previously scarce and costly [26]. This pattern reflects a broader global dependence on horse-derived immunoglobulins for treating tetanus, rabies, botulism, and envenoming by snakes and other venomous animals, particularly in tropical [27], resource-limited settings where snakebite is recognized as a neglected public-health problem by the World Health Organization [28]. However, the welfare of horses maintained for biomedical plasma production warrants specific attention. The Suoi Dau facility, operated by IVAC under Vietnamese state regulation, maintains comparatively controlled conditions, with horses subject to centralized veterinary oversight, defined collection intervals, and monitored body condition. Nevertheless, independent welfare assessments of production horses at this and similar facilities have not been published, and the integration of standardized welfare indicators into serum-production monitoring protocols would represent meaningful progress.

The co-occurrence of biomedical, cultural, and livelihood functions within a single species is unusual in the context of Vietnamese livestock and reflects a degree of social embeddedness. This deep integration into community life may confer resilience to the economic pressures (mechanization, rural outmigration, and changing land use) that have depleted horse populations elsewhere in Southeast Asia. Therefore, conservation investment in Vietnamese horses carries returns across health, heritage, and rural development simultaneously, making the case for coordinated policy attention stronger than population size alone would imply.

## 5. Progress in Equine Research in Vietnam

Equine research in Vietnam has developed more slowly than research on pigs, poultry and ruminants, and much of the available information is embedded in grey literature, internal reports and provincial documents rather than international journals. Nevertheless, several important aspects can be distinguished, including work on breeding and crossbreeding, conservation of genetic resources, reproductive management, health and parasitology, welfare of working and biomedical horses, and the emerging field of sport and tourism horses. Together, these strands reveal both significant technical progress in particular niches, especially at state breeding and research centres, and large, persistent knowledge gaps in smallholder systems and socio-economic dimensions.

### 5.1. Breeding, Selection and Genetic Improvement

Genetic improvement for horses has significantly improved across the world, especially with support from accurate phenotyping [29] and inclusion of genomic information [30]. However, modern quantitative genetic and genomic selection methods, including estimated breeding values (EBVs), BLUP-based selection indices, and SNP-based genomic evaluation methods, have not been applied to Vietnamese horses. Indigenous horses in Viet Bac and other upland regions have been small-framed but sure-footed animals, well-adapted to steep terrain and low-input feeding conditions. However, their limited body size and traction power constrain load capacity and speed, especially as road infrastructure improves and demand for heavier transport increases [31]. To address this, state breeding centres have introduced improved genotypes and implemented structured crossbreeding schemes over several decades [32]. One major initiative involved the use of Kabardin horses, a hardy mountain breed from the Caucasus, crossed with local Vietnamese mares to produce animals combining greater body size and strength with good adaptation to mountain conditions. The primary objective of crossbreeding with Kabardin blood was to increase load-bearing capacity and body weight in pack horses used for agricultural transport and military logistics while retaining the sure-footedness and low-input resilience of native animals. Programs at the Center for Animal Breeding Research and Development in Mountainous Areas have used mating schemes yielding crossbreds with approximately 25% Kabardin blood, which provides a compromise between growth and adaptation traits [31]. Field reports indicate that these crossbreds exhibit increased height at withers, longer stride, improved traction and greater carrying capacity, while maintaining sure-footedness and suitability for pack work in the northern provinces [31]. More recently, research projects have explored the creation of sport and tourism horses by further crossing Kabardin-derived stock with high-performance European breeds.

The crossbreeding history of Vietnamese horses extends over more than a century. As early as 1893, twenty-two Bretonne and Lande stallions imported from France were crossed with local mares, producing offspring that retained native hardiness while achieving greater body size and work capacity, though research was discontinued due to unclear objectives [33]. In 1906, 275 French mares and 200 Australian mares were introduced at stations in Cao Bang, Thanh Hoa, An Khe, Hue and Gia Dinh; crossbreeding between native stallions and Australian mares at An Khe and Hue produced quarter-blood offspring with improved height of 128–130 cm at the withers. Arab stallions imported from Bombay in 1916 yielded three-quarter-Arab crosses reaching 145 cm and five-eighths-Arab crosses reaching 135 cm at the Tan Son Nhat stud in Saigon [33]. Systematic state-led crossbreeding began in earnest following the import of eight Kabardin horses (five stallions, three mares) from the Soviet Union in June 1964 for adaptive trials at Ba Van, Thai Nguyen, with crossbreeding research commencing during the 1970s. A further three Kabardin animals were imported from Heilongjiang, China, in February 2000 for genetic refreshment of the breeding line. At 36 months of age, these animals recorded a body weight of 450–500 kg, withers height of 155 cm, oblique body length of 160 cm, girth of 183 cm, pack capacity of 120 kg, and a racing speed of 35 km/h [33]. Crossbreeding trials established that the 25% Kabardin formula was most appropriate for smallholder mountain farming conditions. At 24 months, 25% Kabardin crossbreds reached 310 kg body weight in Bac Ha, Lao Cai [34]. The combined population of such crossbreds in Ha Giang, Lang Son, Lao Cai and Yen Bai exceeds 20,000 animals, 17.86% of the national herd, with withers height of 125–128 cm, body weight of 238–246 kg, draft capacity of 900–1000 kg and pack capacity of 70–80 kg [35]. Between 2006 and 2010, 72 semen doses from Westphalian and Oldenburg stallions were imported from Germany for crossing with Kabardin-cross mares at the Mountain Livestock Research and Development Centre. The resulting three-blood crossbreds (10 colts, 12 fillies) recorded birth weights of 30.43–31.78 kg, body weight of 326.66–348.78 kg at 24 months, and racing performance of 43.75 km/h over 1000 m and 39.68 km/h over 1500 m [36].

In parallel with crossbreeding for improved work and sport performance, Vietnamese institutions have also recognized the need to conserve native equine genetic resources [11,37]. Ba Van Hill has been described as one of the country’s largest livestock gene pools, housing horses and buffalo along with other species. Within this framework, local horse lines and special colour variants, such as white horses and miniature horses, have been maintained for both conservation and economic purposes. White horses, for instance, have potential value for tourism, ceremonial functions and sport, while miniature horses are attractive for leisure and children’s tourism activities. Despite this, equine conservation remains much less developed than conservation programmes for pigs [38,39], cattle [40,41] and poultry [42,43]. There is little published work on the genetic diversity of Vietnamese horse populations, and no comprehensive molecular characterization of local, Kabardin-cross and three-blood sport horses has been reported in the international literature. This stands in contrast to the relatively advanced status of genomic studies in other Vietnamese livestock species, where conservation of indigenous breeds has been analyzed in detail. For horses, conservation activities are still largely phenotypic and population-based: maintaining small nucleus herds, avoiding extreme inbreeding within centres, and monitoring basic performance traits. From the perspective of global animal genetic resources management, this represents both a vulnerability and an opportunity because the existing nucleus herds at Ba Van and similar centres could be the starting point for robust genetic and genomic characterization to inform conservation and future breeding strategies [37]. From a global perspective, the Vietnamese equine nucleus herds represent a genetically distinct population within the broader Southeast Asian pony complex, and their molecular characterization would contribute to international efforts to document and conserve equine genetic diversity.

### 5.2. Reproductive Management and Physiology

Reproductive management is a crucial component of equine production, affecting both the number and the quality of foals produced and the efficiency of breeding programmes. In Vietnam, however, published data on reproductive parameters in horses are scarce, and most available information comes from internal reports and technical guidelines at research centres. These documents indicate that both natural service and, to a limited extent, artificial insemination are employed in structured breeding programmes. In smallholder mountain systems, stallions are typically owned by households or villages and used for free mating during the breeding season, with limited recording of service dates or reproductive outcomes. Age at first foaling may be relatively late compared to improved equine systems, reflecting nutritional constraints and limited reproductive management, while foaling intervals are likely prolonged by seasonal feed shortages and poor body condition. Native Vietnamese horses and 25% Kabardin crossbreds age at first estrus has been reported at 20–22 months, estrous cycle length at 21–23 days, estrous duration at 7–9 days and gestation length at 330 ±15 days [44]. Postpartum return to estrus occurs within 9–12 days, a shorter interval than most other domestic species, reflecting the absence of a prolonged luteal phase at parturition in mares [44]. For the White Horse specifically, age at first estrus averages 20.83 months (±SD not reported in primary source), estrous cycle length 21.52 days (range 19–24 days), estrous duration 7.78 days (range 6–10 days) and gestation length 328.52 days (±11 days). Age at first foaling averages 34.97 months, and postpartum estrus return averages 10.43 days. In ten *in situ* and two *ex situ* conservation models monitored from 2011 to 2014, the estrous detection rate among 128 monitored mares averaged 82.81%, conception rate 96.68%, foaling rate 95.25% of confirmed pregnancies and foal survival to weaning 92.80% [45]. These figures, while encouraging, derive from closely managed research herds and are likely to overestimate performance in typical smallholder conditions. Optimal mating timing has been identified as day 5–7 of estrus, corresponding to the period immediately preceding ovulation. Palpation and ultrasonographic monitoring of follicular development, as routinely practiced at Ba Van, allow more precise timing but are not accessible to smallholder producers. Extending basic estrus detection and mating record-keeping to provincial livestock stations and farmer groups would represent a cost-effective first step toward improving reproductive efficiency across the national herd [44,46].

### 5.3. Health, Parasitology and Welfare

The most clearly documented strand of equine health research in Vietnam concerns gastrointestinal parasites in horses in the northern provinces [47]. The study reported that strongyle infections are highly prevalent among adult horses used as pack and farm animals, with mean faecal egg counts around 2053 eggs per gram in packhorses from mountainous areas and many animals exceeding 500 eggs per gram. The study highlighted that deworming practices were irregular or absent in many smallholder systems, with owners often unaware of the impact of helminths on weight, endurance and general condition. The authors suggested that targeted anthelmintic programmes could significantly improve the health and performance of working horses but also warned about potential anthelmintic resistance if treatments were not carefully managed. Beyond strongyles, Vietnamese horses likely harbour a range of other parasites and infectious agents, including ascarids in foals, tapeworms and possibly vector-borne diseases such as piroplasmosis, given the presence of suitable tick vectors in the region. However, systematic surveillance data are scarce, and equine diseases rarely appear as a separate category in national epidemiological reports, which focus on major production species and zoonoses. Notably, the first molecular confirmation of *Theileria equi* in Vietnamese horses was reported by Dao et al. (2026), providing direct evidence that piroplasmosis poses a genuine surveillance and control challenge in the region [48].

It is worth noting that Vietnam is an active member of the World Organisation for Animal Health (WOAH) and fulfils reporting obligations at both national enforcement and international notification levels. However, horses are rarely the primary focus of these reports, which concentrate on major production species and recognized transboundary diseases. This lack of visibility may create blind spots in disease control policy, especially as horse movement for tourism and trade increases. There is also little public information on vaccination practices for horses, apart from occasional references to tetanus and rabies prophylaxis in general livestock extension documents.

State veterinary records and research station reports document several bacterial diseases of concern in northern mountain provinces. *Haemorrhagic septicaemia* caused by *Pasteurella multocida* serotypes B2 and E2 occurs in horses in proximity to affected buffalo and cattle herds in provinces including Lao Cai, Yen Bai and Son La, with clinical signs including acute fever (41–41.5 °C), respiratory distress and death within 6–24 h in peracute cases [33]. Equine pneumonia attributable to *Streptococcus equi*, *Diplococcus pneumoniae* and *Actinobacillus pleuropneumoniae* has been associated with significant mortality in northern mountainous districts: between 1996 and 2000, 364 horses died of infectious pneumonia in Bac Ha, Lao Cai province alone, and over 200 died in Mu Cang Chai, Yen Bai province between 1997 and 1999 [33]. These outbreaks highlight the importance of establishing routine vaccination programmes and improving veterinary diagnostic capacity at the provincial level.

Trypanosomiasis caused by *Trypanosoma evansi* (Surra) is a recognized vector-borne disease of horses in Vietnam, transmitted principally by biting flies (*Tabanus* and *Stomoxys* spp.). Clinical progression includes intermittent fever (40–41 °C), progressive weight loss, peripheral oedema, anaemia and neurological signs leading to death within 1–6 months if untreated. Control depends on biannual prophylactic treatment with Naganol or Trypamidium, combined with vector management and pre-movement screening. Blood parasite infections, more broadly, if anthelmintic or antiprotozoal prophylaxis is not maintained, can affect 26–30% of animals in acute and chronic forms; two prophylactic injections per year reduce prevalence to below 5% [49]. The welfare of working equids has received increasing international attention, with standardized tools such as the Standardised Equine-Based Welfare Assessment Tool (SEBWAT) and the Equid Assessment, Research and Scoping tool (EARS) now applied across multiple low- and middle-income countries [50,51]. However, in Vietnam, working-equid welfare has only sporadically appeared in scientific publications or policy documents. Reports from mountainous regions of Vietnam describe horses carrying heavy loads over long distances on steep, rocky paths using simple harnesses and with limited hoof care, conditions that mirror those associated with lameness, back injuries, skin lesions and chronic fatigue in working equids elsewhere in low-resource settings [51]. At present, there is no standardized welfare assessment protocol for working horses in Vietnam comparable to those applied in some other low- and middle-income countries by NGOs and research groups [52]. Incorporating welfare indicators such as body condition scoring (BCS), lesion scoring, lameness, and behavioural signs of distress into existing research and extension programmes would provide an evidence base for improving husbandry conditions without undermining livelihoods and represents one of the most actionable near-term research priorities for the sector.

## 6. Priority Areas for Future Equine Research and Development in Vietnam

The priority areas for future equine research and development should focus on genetics, reproduction, health and welfare, socio-economics and knowledge systems (Table 3).

### 6.1. Genetic Characterization and Conservation

Genetic characterization of native horse breeds is a globally recognized conservation priority; Vietnamese horse populations, as yet uncharacterized at the molecular level, represent an understudied component of worldwide equine diversity that may carry unique alleles relevant to disease resistance, altitude adaptation and metabolic efficiency. A main priority is the accurate genetic characterization of Vietnamese horse populations, including local mountain horses, Kabardin-cross work horses, and emerging three-blood sport horses [36,45,53,54]. Nationwide genomic surveys using microsatellites or SNP panels should be conducted to describe genetic diversity, population structure, and relationships among regional populations and farm types, similar to other program have been done worldwide [55,56]. The research can prioritize herds concentrated in northern mountainous provinces, where horses remain central to ethnic minority livelihoods [57]. Based on these data, researchers can estimate effective population sizes, inbreeding coefficients, and genetic erosion risks, especially in small, isolated populations and intensively selected lines [58]. This information can be used to establish conservation herds and community-based breeding programmes that maintain variation while improving key traits such as work capacity, health, and temperament [59]. Parallel efforts should focus on developing formal breed descriptions and registration systems for distinct types (e.g., local upland horses, Kabardin-cross draught horses, three-blood sport horses, white and miniature horses), which would support both conservation and branding for tourism and niche markets [1].

### 6.2. Reproductive Biology and Breeding Management

Reproductive biology and breeding management under Vietnamese production conditions remain largely undocumented in the scientific literature and should be the main focus [54]. It is necessary to perform a systematic evaluation of fertility, foaling rates, and seasonal breeding patterns across major production systems. These kinds of studies should include smallholder mountain herds, state breeding centres and private sport-horse farms. These studies should record age at first foaling, foaling interval, conception rates under natural service versus artificial insemination, and perinatal losses over multiple years, allowing identification of system-specific bottlenecks. Using such data, breeding strategies can be refined to enhance reproductive efficiency while controlling inbreeding, for example, through structured mating plans, stallion rotation schemes, and the development of selection indices that combine reproductive performance with work or sport traits. Research should also assess how nutritional status and management differ between high-altitude and low-altitude environments and how these differences influence reproductive performance, enabling the design of feeding and body-condition targets for mares and stallions in different agro-ecological zones. In vitro reproductive technologies for conservation should also be prioritized [60].

### 6.3. Health, Nutrition and Welfare Studies

Comprehensive studies on health, nutrition, and welfare are essential to underpin any sustainable expansion of the equine sector, particularly in smallholder systems where baseline data are almost absent [61]. A first step is to establish national baseline disease-prevalence data for key conditions, including strongyle and other gastrointestinal parasites, tick-borne infections such as equine piroplasmosis due to *Theileria equi* and *Babesia caballi*, respiratory diseases, and musculoskeletal problems [62]. Standardized sampling and diagnostic protocols should be applied across northern, central, and southern regions and across farm types to generate comparable data that can guide targeted interventions. Based on these baselines, vaccination and deworming schedules tailored to local conditions can be developed and tested, taking into account parasite epidemiology, climate, management practices, and the risk of drug resistance in smallholder systems where current anthelmintic use is low and unsystematic [62]. Furthermore, vaccination programmes targeting viral respiratory pathogens and *Clostridium tetani* should be prioritized within national health strategies, as tetanus remains a significant occupational risk for working horses in smallholder systems where wound exposure during agricultural labour is frequent. Parallel research is needed to create nutritional-requirement models for indigenous and crossbred horses under typical Vietnamese feeding regimes, including grazing, crop residues, and agro-industrial by-products. These models should underpin practical ration recommendations that support work performance, growth, and reproduction without high cost and should be validated on-farm through productivity and body-condition responses. Welfare assessments must be integrated into health and nutrition work, particularly for working horses in mountainous areas and tourism horses at Bá Vân and other emerging destinations, using validated indicators such as body condition, lameness, skin lesions, harness fit, workload, and behavioural signs of distress [63]. Such assessments will help identify priority welfare problems and inform interventions, ranging from improved harness designs and rest schedules to owner training, that can be evaluated for both welfare outcomes and livelihood impacts.

### 6.4. Economic and Market Analysis

Economic and market-oriented research is needed to quantify the contribution of horses to rural livelihoods [54]. It might also need to identify promising value-chain opportunities within Vietnam’s transforming livestock sector. Therefore, studies should estimate the economic value of horses in transport, agricultural work, and tourism, including both direct cash income and indirect benefits such as reduced labour time, improved market access, and enhanced resilience for smallholders in remote areas. This requires farm- and household-level data on costs, revenues, and time budgets collected across representative regions, particularly in northern mountainous provinces where horses remain integral to ethnic minority economies. Moreover, value-chain analyses can then map existing and potential markets for equine services and products, including tourism rides, carriage services, racing, specialty products, and biomedical derivatives from donor herds at Ba Van. Consequently, these results help to identify bottlenecks in input supply, service provision, processing, marketing, and regulation. These analyses should also examine labour-saving innovations, including small-scale mechanization and improved road infrastructure, that may complement rather than abruptly displace equine roles in remote areas, helping to design culturally sensitive transition pathways that respect local preferences, protect the most economically vulnerable households from unintended negative effects, and preserve the social and ecological knowledge systems that communities have developed around horse management over generations.

An important gap in Vietnam’s equine sector is the absence of formal education pathways for equine practitioners. Developing accessible training materials in Vietnamese covering nutrition, hoof care, harness design, parasite management, and welfare assessment and establishing links with regional equine education providers would significantly strengthen the human capital base underpinning sustainable sector development.

Ethnoveterinary knowledge held by ethnic minority communities and long-term horse owners represents a rich but systematically under-documented resource. This knowledge is also an insufficient resource for equine health and management in Vietnam. Priority research should systematically document herbal treatments, traditional disease classifications, husbandry rules, and local criteria for assessing horse health and suitability, using participatory rural appraisal and local language documentation to capture this knowledge accurately and respectfully. Subsequent studies should evaluate the safety and efficacy of widely used medicinal plants and practices through pharmacological testing and carefully designed field trials, building on international examples where ethnoveterinary remedies have been validated or refined.

A notable result emerging from this synthesis is that most quantitative equine data in Vietnam derive from state breeding centres, particularly Ba Van. It is important to note that this concentration of institutional knowledge might have influenced policy and breeding decisions in ways that do not reflect the health, reproductive, and welfare realities of the national herd. Therefore, further studies integrating on-farm data collection across diverse production systems are required for the development of a robust and representative evidence base.

### 6.5. Capacity Building and Extension Services

Compared to other livestock, under-investment in equine research institutions and current gaps in disease management underscore the need for capacity building at multiple levels [64]. It is important for re-establishing dedicated equine research capacity by increasing investment in facilities, diagnostic laboratories, equipment, and human resources. Veterinary education programmes should integrate equine medicine more systematically into curricula and offer continuing-education modules for practicing veterinarians working in horse-keeping areas [65].

Moreover, extension programmes tailored to equine health and management need to be developed and deployed in remote mountainous regions where horses remain central to livelihoods [66]. These programmes can disseminate practical guidance on nutrition, parasite control, hoof care, harness design, and welfare. Supporting the formation and strengthening of farmer organizations and cooperatives focused on horses can facilitate collective action for disease control, joint purchasing of inputs, and improved market access, building on broader livestock cooperative experiences documented in Vietnam. It is also important to engage with regional networks and international partners for access to specialized expertise, training opportunities, and collaborative research projects. These engagements and collaborations might help Vietnam align its equine sector with emerging global standards and innovations.

## 7. Limitation

Nevertheless, several limitations of this review should be acknowledged. Firstly, the available evidence base for Vietnamese equine science is narrow and geographically concentrated, with the majority of quantitative data deriving from institutional reports, provincial veterinary records, and Vietnamese-language publications that have not undergone international peer review. This may have introduced reporting bias toward findings from state breeding centres, particularly the Mountain Livestock Research and Development Centre at Ba Van, while smallholder systems in more remote provinces remain poorly represented in the literature. Furthermore, the absence of a systematic database search protocol was necessitated by the fragmented and largely non-indexed nature of the Vietnamese equine literature. That means that relevant studies published in Vietnamese-language journals or institutional repositories may have been inadvertently omitted. It is important to note that references include grey literature for key quantitative claims, including population estimates, disease mortality figures, and crossbreeding performance data, which might have influenced the precision and generalizability of the findings presented here. Yet, this review represents, to our knowledge, the first English-language synthesis of available evidence on equine knowledge in Vietnam. The limitations identified here themselves constitute a strong justification for investing in peer-reviewed, systematically recorded equine research in the country. Further studies integrating standardized on-farm recording, molecular genetic characterization, and nationally coordinated disease surveillance are required for the development of a robust and verifiable evidence base for Vietnam’s equine sector.

## 8. Conclusions

Vietnam’s horse population, though small, represents a genetically and culturally distinct component of global equine diversity. It supports upland livelihoods, sustains ethnic minority traditions, and serves niche biomedical and tourism markets. This review provides the first English-language synthesis of Vietnam’s equine history, production systems, health, and socio-cultural roles, consolidating information that has been scattered and difficult to access. Our assessment highlights Vietnam’s long-standing institutional commitment to conserving native horses and identifies clear priorities for future research and development, including genetic characterization, reproductive management, health surveillance, welfare, and capacity building. Conserving these breeds contributes to global equine diversity and offers insights into adaptation to tropical mountain environments. Strengthening coordinated, evidence-based efforts will help Vietnam enhance the resilience and sustainability of its equine sector while preserving its unique genetic and cultural heritage.

## Figures and Tables

**Table 1:** Horse breeds or populations in Vietnam and their key features.

Breed/Type	Main Distribution	Typical Role	Height at Withers (m)	Key Adaptations	Cultural Association	Breed Origin and Genetic Composition	References
Muong Long	Muong Long, Ky Son (Nghe An)	Pack transport on steep highland trails	1.1–1.3	Cold, foggy, karst terrain; low inputs	Strongly linked to the Hmong of Muong Long	Autochthonous local population	[10]
Hmong	Northern upland provinces (e.g., Ha Giang)	Pack transport, riding	1.1–1.3	Rugged terrain; low-quality forage	Hmong and other northern ethnic groups	Local mountain pony type	[9]
Vietnamese White	Mountains of northern Vietnam	Pack work; ceremonial and tourism	1.1–1.3	Mountain climate; coat depigmentation	Local cultural and symbolic value	Variant of local pony stock	[11]
Kabardin-cross	Research farms; northern uplands	High-capacity pack, draft, and sport	1.2–1.6	Tolerant of mountain terrain; improved work and sport performance under structured management.	Development programs, racing, and sport	25% Kabardin crossbred	[1]

**Table 2:** Functions of Horses in Vietnamese Smallholder Farming Systems.

Function	Description	Economic Contribution	Current Trend
Pack transport [13]	Carrying crops and goods	Reduces hired labor	Still essential in remote areas
Farm support [17]	Light draught and hauling	Seasonal utility	Declining
Household asset [17]	Store of value	Emergency sales	Stable
Cultural use [4]	Festivals, markets	Community identity	Increasing importance

**Table 3:** Proposed Research and Development Roadmap.

Priority Area	Key Activities	Expected Outputs	Long-Term Impact
Population Assessment	National census, mapping of distribution	Reliable demographic database	Evidence-based planning
Genetic Conservation	DNA characterization, identification of local types	Conservation guidelines	Protection of indigenous resources
Animal Health	Disease surveillance, extension training	Improved veterinary knowledge	Healthier and more productive animals
Production Systems	On-farm performance studies	Management recommendations	Sustainable utilization
Socio-cultural Integration	Link horses with tourism and heritage programs	Community-based initiatives	Economic incentives for conservation
Animal Welfare	Baseline welfare assessment, development of national welfare standards, owner training	Standardized welfare protocols	Improved equid wellbeing and livelihood sustainability

## Data Availability

No new data were created or analyzed in this study. Data sharing is not applicable to this article.
